
JOURNAL INFORMATION
==============================
NLM Title Abbreviation: Tex Med J (Austin)
NLM Journal ID: 21130590R

Texas Medical Journal

ISSN: 0892-8495
Publisher: Biomedical Journal Digitization Project

ARTICLE INFORMATION
==============================
PMCID: PMC9625904
Article version: 1
Subjects: Books and Magazines

Text-Book of Materia Medica for Nurses

Print publication date: 1906 Jan
Volume: 21
Issue: 7
First page: 257
Last page: 257
License: This work is free of known copyright restrictions. For more information, please see PMC Back Issue Digitization.
License URL: https://creativecommons.org/publicdomain/mark/1.0/

==============================

---

Text-Book of Materia Medica for Nurses. Compiled by Lavinia L. Dock, Graduate of Bellevue Training School for Nurses. Fourth edition, revised and enlarged. G. P. Putnam's Sons, New York and London. The Knickerbocker Press, 1905. Cloth, 320 pages. Price, $2.

Trained nurses for every kind of injury, illness and operations, and for obstetrical cases, are now a recognized necessity, and no conscientious practitioner will dispense with one. Hence, schools for their education and training are springing up in every large city, and text-books for their use are as indispensable as they are for the physician. Miss Dock has done a good work and supplied an evident want in the publication of the little book. Of course, nurses must be familiar with the materia medica and should be familiar with therapeutics. The publishers have gotten it out in their usual excellent style. We commend it to the attention of those who are interested, especially in instructing and supplying nurses. D.

---

### Publisher's Department.

Heavy Colds.—The rheumatic and grippy conditions which so frequently accompany heavy colds are sometimes overlooked. By the prompt use of Tongaline the irritating features of these conditions are ameliorated and the congestion is relieved, while the
